# Artificial intelligence in transitional care: practice, promise, and pitfalls—a scoping review

**DOI:** 10.3389/fdgth.2025.1690223

**Published:** 2026-01-26

**Authors:** Amal Fakha, Albert Boonstra

**Affiliations:** Department of Innovation Management and Strategy, Faculty of Economics and Business, University of Groningen, Groningen, Netherlands

**Keywords:** AI applications, AI tools, artificial intelligence, care coordination, care fragmentation, care transitions, care continuity, transitional care

## Abstract

**Background:**

Care transitions, which involve the movement of patients between different care settings are critical moments in the care continuum but are often compromised by fragmented care delivery or poor information transfer among providers. To address this, Transitional Care (TC) programs were developed to address these challenges. Recently, Artificial Intelligence (AI) tools were introduced to support and streamline care transitions. However, their use in TC remains underexplored, highlighting the need to better understand their potential to optimize patient care and reduce adverse outcomes. This review aims to identify the current AI tools applied in TC, their usage to either prevent or improve care transitions, and their associated outcomes.

**Methods:**

A scoping review was conducted following the Arksey and O'Malley framework. Web of Science, PubMed/MEDLINE, and IEEE Xplore were the searched databases, and eligible studies published between 2013 and 2025 were retrieved. Data were extracted from the included studies and mapped to the established categories of AI usages, as well as the eight components of comprehensive TC model. In addition, reported outcomes on the impact of AI on TC were retrieved.

**Results:**

Out of 211 studies identified, 21 were included. The retrieved twenty-one AI tools aimed at enhancing care transitions mostly from hospital to home settings. The majority of the AI tools were used to enhance TC by improving discharge planning, follow-up care, interoperability and system navigation. The components of comprehensive TC mostly promoted by AI tools were care continuity, complexity management, and patient and caregiver well-being. Patient engagement and education were the components least promoted by AI tools. Reported outcomes included rehospitalization rates, earlier prediction and diagnosis, and information exchange.

**Conclusion:**

AI tools for TC are used to enhance care coordination, serving as a catalyst for delivering high-value care. Their application to care trajectories between multiple settings shows a promise for streamlining transitions and fostering patient engagement. However, although challenges lie in integrating these AI tools into clinical decision-making processes and workflows, they hold significant promise for enhancing TC.

## 1 Introduction

Transitional care (TC) is a critical component of healthcare delivery. It is defined as the provision of time-limited care services to ensure patient care continuity by promoting their safe and timely transfer between different levels of care or types of care settings (1). These care transitions are vital for patients with chronic diseases and multimorbidity, who often require care from healthcare providers across various settings (2, 3). Consider, for instance, a stroke survivor who is transitioning from hospital to rehabilitation services (4) or an older individual with chronic conditions who receives interdisciplinary care (5). Similarly, adolescents and young adults with childhood-onset disabilities can undergo diverse care transitions across various healthcare providers to obtain the care needed (6). Hence, being affected by long-term or complex health conditions requires care services from multiple care settings, resulting in more care transitions, such as but not limited to hospital to home, hospital to rehabilitation, or home to nursing home (5, 7).

These transitions, while essential, are vulnerable phases for patients, often plagued by care fragmentation, lack of coordination, and poor communication among healthcare providers (8, 9). Consequently, patients may experience adverse outcomes such as medication errors, hospital readmissions, or even mortality (10). Therefore, to circumvent the challenges of care transitions, numerous TC models, programs, and interventions (referred to as TC throughout this paper) were developed and implemented to either ensure care continuity throughout the transitions between different healthcare settings or to prevent care transitions by providing the care needed in order to avert unnecessary or avoidable transfers (11, 12). Critical components of TC include but are not limited to forming multidisciplinary care teams, appointing transition care managers or nurses, fostering patient self-management, structuring the care process, and ensuring patient follow-ups at home (12). Moreover, shared decision-making involving informal caregivers and the patient's family can be used to establish an individualised care plan to improve care transitions (13). Likewise, Naylor et al. (14) identified eight components of effective TC necessary to ensure optimal care transitions, such as patient and caregiver engagement, complexity management, care continuity, and patient/caregiver education.

Therefore, implementing TC plays a significant role in decreasing rehospitalisation, reducing the number of emergency department visits, and ensuring a safe transfer between healthcare settings such as nursing facilities or rehabilitation centers to home and vice versa, as well as between various other care transition pathways (13, 15). Furthermore, research has shown that if continuity of care is ensured, it will result in enhanced access to services, reduced hospitalisations, increased patient satisfaction, and improved patient health outcomes (7). Nevertheless, creating an interdisciplinary, coordinated transitional care environment remains a challenging endeavour (7, 16). Thus, enhancing the quality and safety of care transitions is a top priority for the healthcare systems of many countries (17).

With the continuous advancement of Artificial Intelligence (AI) in recent years, there is a growing trend towards integrating this innovative technology into daily healthcare practices, including TC. In this paper, we use the term AI to describe computer-based tools that support human decision-making by recognizing patterns, making predictions, or generating language. This includes both rule-based systems and newer technologies like large language models (LLMs) and generative AI (GenAI). These tools are trained on large datasets—such as electronic health records (EHR) and clinical guidelines—and work by identifying patterns in the data to suggest likely outcomes (18, 19).

AI offers promising solutions to alleviate the shortage of healthcare professionals and meet the patients' rising demands for high quality, timely, and efficient care delivery (20). In the context of TC, new opportunities have emerged for employing AI to streamline patient discharge processes, perform patient triage, facilitate information exchange, and support clinical decision-making (21). Enabled by advances in data integration and machine learning, AI has the potential to transform healthcare by automating routine processes, identifying patterns and generating insights to support care-teams (22). While AI is often defined as the ability of machines to perform tasks associated with human intelligence, such as reasoning, learning, planning, and communication (23), its practical application in healthcare is largely focused on supporting clinicians, reducing administrative burden, and enhancing patient outcomes, rather than replicating or replacing human cognition. Thus, the overarching goal of AI in healthcare is to augment healthcare delivery by enabling safer, more efficient, and more coordinated care. More specifically, AI encompasses systems designed to support healthcare professionals in clinical decision-making, diagnostic accuracy, and personalized treatment planning, ultimately leading to more efficient and effective patient care (24).

Recently, AI has been used for TC to improve human decision-making and increase effectiveness (25). Given that TC is a uniquely complex type of care delivery, involving multiple settings and diverse healthcare providers posing challenges such as communication gaps, fragmented care plans, and increased risks of errors; AI is particularly well-suited to address this, as its capabilities align closely with the needs of TC (8, 9). Hence, for patients undergoing care transitions, AI can help ensure that patients' needs are considered and realised. For example, AI-powered tools can support the patient discharge process from emergency departments by automatically deciding on the most fitting type of follow-up care and arranging it, as well as exchanging the patient's medical information between care settings, thus contributing to an enhancement of the delivery of TC (26, 27). Mazza et al. (28) demonstrated that implementing AI in the form of chatbots to gather patient-generated health data, which then can be integrated into each patient's EHR, can enhance the critical 30-day period post-discharge and stimulate more accountability and collaboration between patients and their caregivers. Also, a study conducted by Hayes et al. (26) examined how AI-enabled tools have the potential to scrutinise patient electronic health record data, extract crucial insights, and generate more concise post-visit summaries. Therefore, AI has a high relevance and potential to optimize TC delivery by integrating data and improving accuracy in information exchange, streamlining communication, accelerating diagnostics and triage for timely interventions, and automating administrative tasks that can save resources (26).

On the contrary, there is evidence that AI's ability to enhance TC can be sometimes constrained with potential risks and ethical issues. Findings of one study underlined that using AI tools can reduce accessibility for patients less familiar with new technologies (29). Moreover, algorithmic bias is another critical concern, as AI systems trained on non-representative datasets can exacerbate health disparities such as misdiagnosing or under-treating marginalized populations due to skewed training data (30, 31). Transparency and accountability are equally pressing; as AI models can obscure decision-making processes, leaving healthcare providers and patients uncertain about how recommendations are generated (e.g., an AI-driven misdiagnosis) (30). Thus, considering the AI's explainability and interpretability (the ability to describe how an AI model arrives at its conclusions and the degree to which humans can comprehend the cause-and-effect relationships within the model) are key elements to address the opacity of AI's “black-box” decision-making (30). Similarly, data privacy and informed consent pose additional risks, as AI's reliance on vast patient data increases vulnerability to breaches and raises questions about whether patients fully understand how their data is being used (31). Also, without intentional design, AI can deepen inequities by failing to adapt to local needs, such as overlooking the socioeconomic background of patients in care transitions (30). Therefore, these challenges demand a focus on algorithmic transparency, accountability, data security and privacy, and patient-tailored design to ensure AI's ethical and equitable integration into TC (32).

Despite the growing interest in leveraging AI for TC, a comprehensive overview of its use and associated outcomes is missing (27, 33). While there are separate studies exploring specific AI applications in TC, no prior work has provided a holistic synthesis of the field. Additionally, although, there is literature examining TC for vulnerable patient groups, the study of implementing AI solutions in this field is still lacking. This study aims to address this gap by examining the current landscape of AI in transitional care, mapping its applications, and identifying its overall impact and potential outcomes associated with its implementation. By exploring the intersection of AI and TC, this study integrates the existing evidence and literature into the first comprehensive overview of the field, and hence contributes to a broader understanding of how this technology can support safer and more effective care transitions in modern healthcare.

## 2 Methods

### 2.1 Study design

The methodology chosen for this study is a scoping review, which adheres to the five steps outlined by Arksey and O'Malley's framework (34), widely recognized as the foundational and established methodology for conducting scoping reviews, and the enhancements proposed in the literature were possible (35, 36). Also, the PRISMA-ScR (Preferred Reporting Items for Systematic Reviews and Meta-Analyses extension for Scoping Reviews) checklist was followed (37) (see Supplementary File 1). So far, little is known about which AI applications exist to be integrated explicitly into TC services (3, 27). A scoping review, therefore, enables a rapid mapping of the key concepts underlying this complex and emerging field of research (34).

#### 2.1.1 Step 1—Identifying the research question

In pursuit of the objectives, the following research question was formulated: What are the current AI tools used for TC, and what are their potential outcomes?

#### 2.1.2 Step 2—Identifying relevant studies

Initially on November 1, 2023, a systematic search of three electronic databases (Web of Science, PubMed/MEDLINE, and IEEE Xplore) was conducted; an update was run on May 25, 2025. Four key concepts were identified to construct a comprehensive search string: applications, artificial intelligence, transitional care, and outcomes. This was based on following the PCC (population, concept, context) framework normally used for scoping reviews in order to guide the inclusion criteria and construct the search string (36). Hence, the PCC helps to map the breadth of available evidence on a particular topic rather than focusing on specific interventions or comparisons. Therefore, the population was identified as the patients during care transitions, the concept was the impact and outcomes of implementing AI for transitional care, and the context was the care settings that involve transitional care services.

### 2.2 Search strategy

To develop a robust search strategy, we first identified the primary keywords for each concept. For each of these keywords, we then generated a list of synonyms, related terms, and variations to ensure that all relevant studies would be captured, regardless of the specific terminology used by different authors. For example, under “Artificial Intelligence,” we kept this term as it is since it is broad enough and includes the concepts of machine learning and deep learning, which allows to capture a large number of relevant studies reporting on the use of AI. As for “Applications,” we considered terms like “implementation,” and “utilization”, and for “Transitional Care,” synonyms and related terms like “care transitions,” “care transfer”, “patient transfer”, and “patient relocation” were utilized. The keyword “Outcome” was used with a truncation (*) in order to explode the search in the database and allow for finding singular and plural forms of the word and the variant endings. Each set of keywords and their corresponding synonyms was combined using the Boolean operator “OR” to encompass all potential variations of each concept. Additionally, controlled vocabulary terms, such as Medical Subject Headings (MeSH) in PubMed/MEDLINE, were employed to ensure a more systematic and exhaustive search, capturing indexed articles that may not use the exact keywords but are relevant. To integrate the four key concepts, the resulting search strings for each concept were then combined using the Boolean operator “AND,” thereby narrowing down the results to studies that address all identified concepts simultaneously. This comprehensive approach ensures that the search is both broad enough to capture diverse studies and specific enough to maintain relevance.

The construction of the search string was a collaborative process among the authors, who carefully discussed and reviewed the different combinations of keywords, controlled terms, and Boolean operators. The final search string was selected based on its ability to maximize the inclusion of relevant studies while minimizing irrelevant ones. Moreover, to further enhance the comprehensiveness of the search, reference lists of all included articles were examined for additional relevant literature that may not have been captured in the initial database search. This manual review helped ensure that no significant studies were overlooked. The complete search strategy, including all keywords, synonyms, and Boolean operators used, is detailed below in Box 1.

Box 1 **Search strategy.**

**Table:** 

Database	Search string used
PubMed	(Application OR Use OR Implementation OR Utilization) AND “Artificial Intelligence”[MeSH] AND (“Transitional Care”[MeSH] OR “Transition of Care” OR “Care Transitions” OR “Patient Handoff” OR “Patient transfer” OR “Patient transition” OR “Patient relocation” OR “Patient handover” OR “Care transfer” OR “Transfer of Care”) AND Outcome*
Web of Science	(Application OR Use OR Implementation OR Utilization) AND “Artificial Intelligence” AND (“Transitional Care” OR “Transition of Care” OR “Care Transitions” OR “Patient Handoff” OR “Patient transfer” OR “Patient transition” OR “Patient relocation” OR “Patient handover” OR “Care transfer” OR “Transfer of Care”) AND Outcome*
IEEE Xplore	(Application OR Use OR Implementation OR Utilization) AND “Artificial Intelligence” AND (“Transitional Care” OR “Transition of Care” OR “Care Transitions” OR “Patient Handoff” OR “Patient transfer” OR “Patient transition” OR “Patient relocation” OR “Patient handover” OR “Care transfer” OR “Transfer of Care”) AND Outcome*

### 2.3 Step 3—Study selection

Literature published between January 1, 2013, and May 25, 2025 was retrieved. Articles eligible for inclusion were those fitting the inclusion and exclusion criteria presented in Table 1. Removal of duplicates from different databases took place. First, the titles and abstracts of the remaining articles were screened for eligibility by a research assistant and under the supervision of the first and second authors. When uncertainty occurred, the research assistant and authors discussed until an overall consensus could be reached. Next, the research assistant and the first author reviewed the full-text articles that were considered suitable individually. Once more, any disagreements and uncertainty were managed via discussion until consensus was found. This approach is consistent with established guidelines for conducting scoping reviews, which prioritize comprehensive coverage and research team consensus-based activity for decision-making and reducing the risk of exclusion errors (36). The PRISMA flowchart was used to describe the study selection process.

**Table 1:** Study eligibility criteria.

Criteria elements	Inclusion criteria	Exclusion criteria
Publication date	Between January 1, 2013 and May 25, 2025	Before January 1, 2013
Language	English	Other than English
Type of publication	Cohort studies, randomised controlled trials, case-controlled studies, case reports, peer-reviewed papers (empirical studies/original research), published conference papers of recognized conferences	Editorials, letters, unpublished graduate theses/dissertations, grey literature, and reviews
Study focus	Studies that describe AI^a^ tools used for Transitional Care^b^ and aimed to improve or prevent care transitions Studies that reported on outcomes (any and not predetermined) to demonstrate the impact of AI tools used for transitional care	Studies referring to either only AI tools or only Transitional Care

a AI refers within this study to computer-based tools designed to perform tasks that typically require human intelligence such as reasoning, learning, planning, and communicating, and moreover to support and enhance healthcare delivery.

b Transitional Care refers within this study to care services implemented to either ensure care continuity throughout the patient transitions between different healthcare providers, settings, departments, and units or to prevent care transitions by providing the care needed in the current setting and avoid unnecessary or avoidable transfers (includes intra- and inter-facility care transitions).

### 2.4 Step 4—Charting the data

Data charting forms were developed and used to retrieve the following data from the included articles:

1. Study characteristics.
2. AI tools: what AI tools exist, and how they are being used in TC.
3. If and how the AI tools can promote a comprehensive TC.
4. Reported outcomes of using AI tools for TC.

The initial data analysis followed a deductive approach (38). First, the retrieved data from the included articles on the existing AI tools used for TC were mapped to the six categories developed by Hayes et al. (26) for AI-enabled tools. These categories in which AI tools are mostly applied include i) discharge/follow-up, ii) triaging/predicting models of care, iii) interoperability, iv) access to mental health follow-up, v) language translation, and vi) system navigation for seniors (26). However, to enhance the clarity, relevance, and analytical rigor of the data analysis, we made deliberate adjustments to these categories. The categories of “interoperability” and “system navigation” were combined into one single category, and the “for seniors” designation was excluded, as system navigation can apply to all patient groups, regardless of age. This decision was based on recognizing that both concepts are intrinsically linked in practice: interoperability enables seamless data exchange across systems, while navigation relies on integrated systems to guide patients through care pathways. Combining these categories eliminated redundancy and better reflected the interconnected nature of these functions in real-world applications. Furthermore, the category “access to mental health follow-up” was integrated into “discharge/follow-up”, since both categories address the continuum of care post-discharge, with mental health follow-up representing a specific subset of broader follow-up care. Thus, consolidating these categories avoided unnecessary fragmentation and emphasized the overarching goal of ensuring continuity of care, regardless of one specific clinical focus. As for the category “language translation” it was retained as a distinct category, whereby it refers to the literal translation into another language or natural language processing which are unique in their focus on overcoming language barriers and facilitating communication. Similarly, the category “triaging/predicting models of care” was kept separate since it refers to early diagnosis, predicting the next appropriate model of care, or anticipating care setting transfer which are functions that are distinct from the other categories. Hence, these adjustments resulted in four mutually exclusive, and collectively exhaustive categories, which strengthened the coherence and conduction of the data analysis and ensured that the findings were both focused and comprehensive. This approach allowed to present a clearer, more actionable way to understand AI applications in transitional care. The four categories used for data analysis are summarized in Box 2.

Box 2 **Hayes’ four categories.**

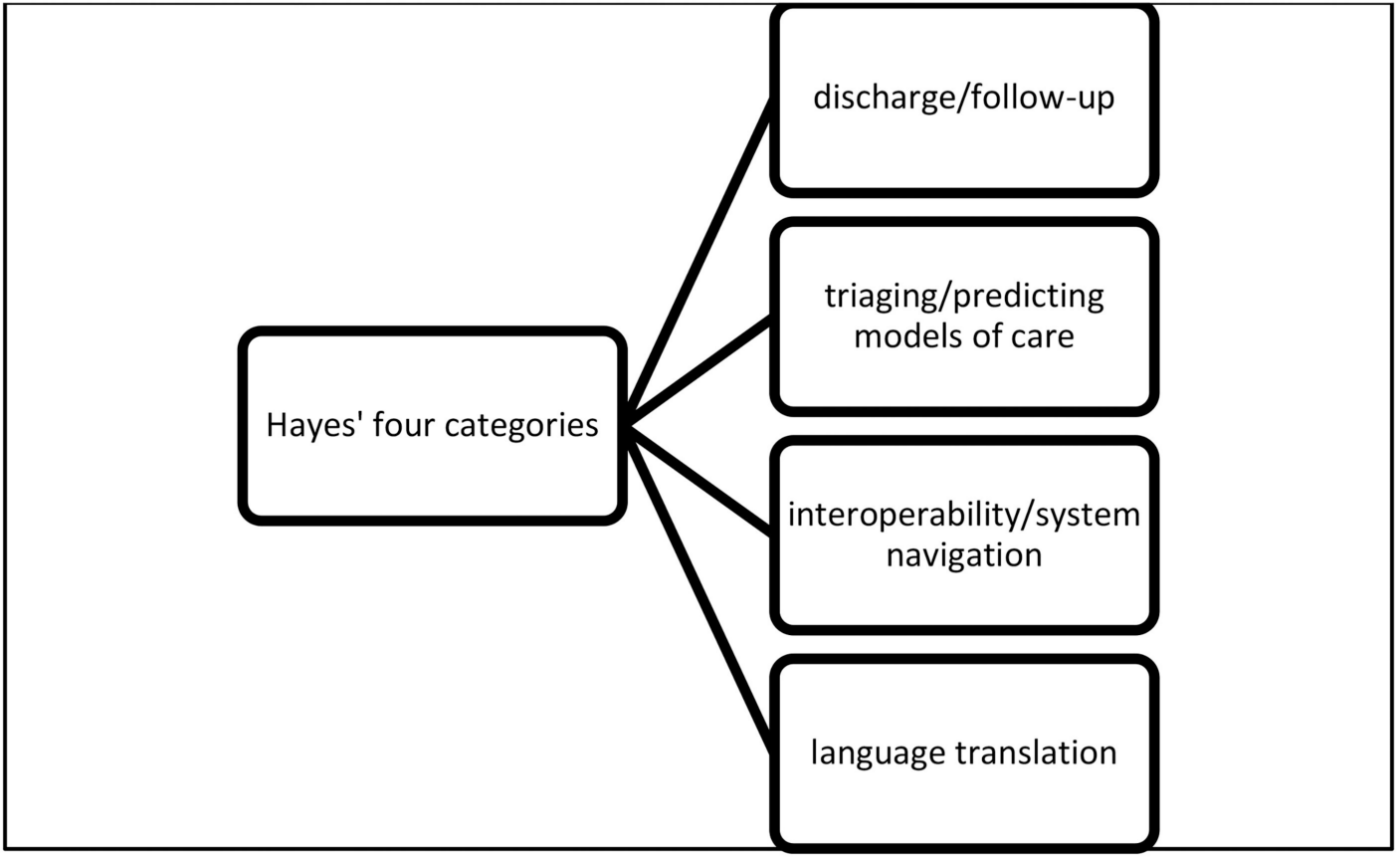

Second, the retrieved data from the included articles on the components of TC that were promoted by use of AI tools were mapped to the eight components of comprehensive and effective TC model developed by Naylor et al. (14). The eight components are patient engagement, caregiver engagement, complexity management, care continuity, patient education, caregiver education, patient and caregiver well-being, and accountability.

A thematic analysis and an inductive approach were also applied to develop in-depth perspectives about AI use in TC and its associated outcomes (39). Hence, inductive coding was used by reading and interpreting data from the included articles to describe and characterize the reported outcomes relevant for the use of AI tools in TC (39).

### 2.5 Step 5—Collating, summarising, and reporting the results

The extracted data were collated and summarised as follows:

1. Description of included studies, including the author(s), year of publication, country, study objective, study design and methods, setting or study population, and study outcomes.
2. Description of the AI tools used in TC by classifying them into four categories developed by Hayes et al. (26), whereas one AI-enabled tool can fit into more than one category.
3. Mapping of the AI tools into the eight different components of the comprehensive and effective TC model developed by Naylor et al. (14). Hereby, one AI-enabled tool can fit into more than one component.
4. Description of the reported outcomes for AI tools used in TC and indicating their frequency across the included articles.

## 3 Results

### 3.1 Study selection

**Figure 1:**
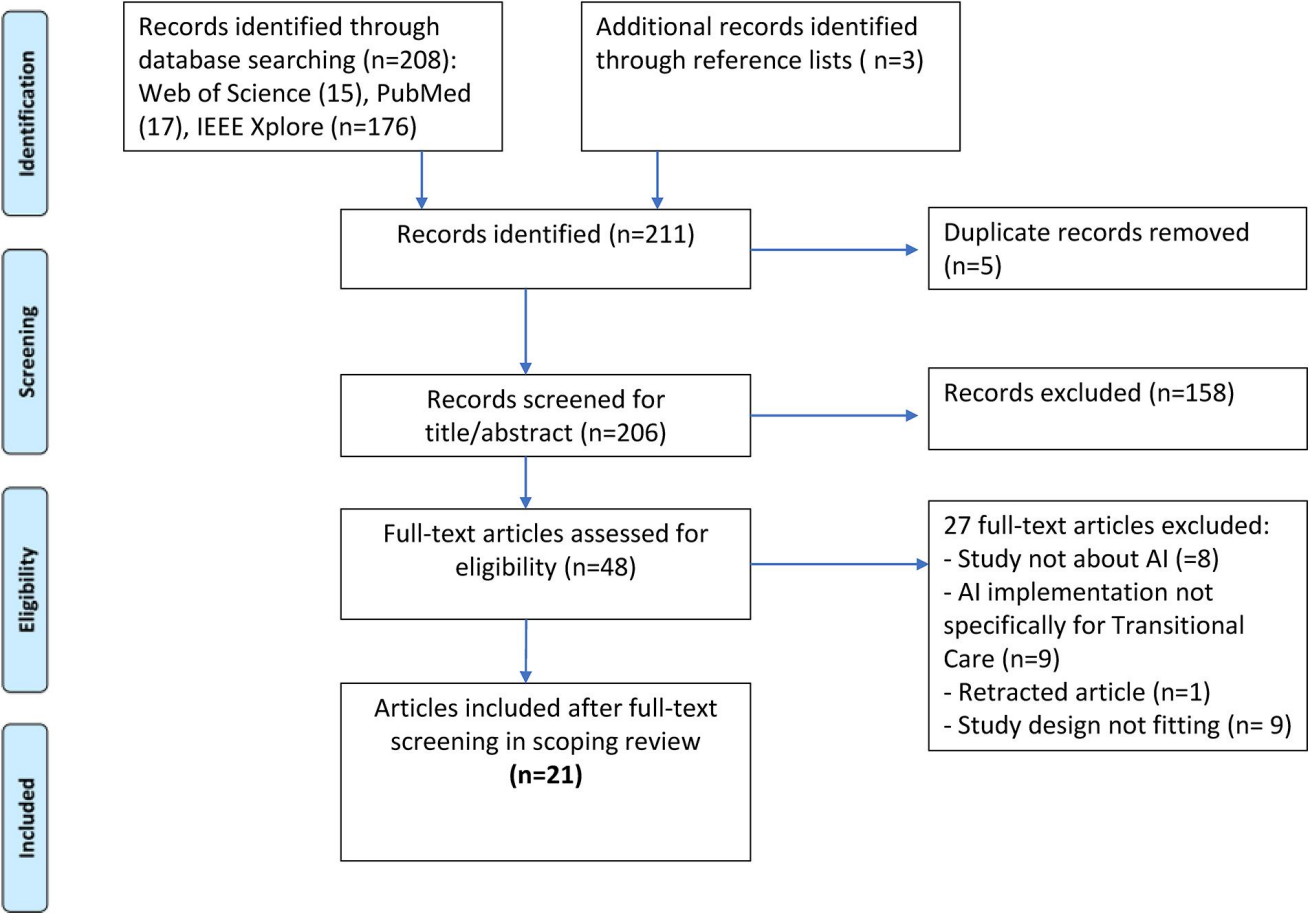
PRISMA flowchart of the study selection process.

Initially, 211 articles were identified, and 21 were included in the final stage. The flowchart for the selection process is depicted in Figure 1.

### 3.2 Study characteristics

Each of the 21 included studies examines the utilisation of an AI tool being integrated into healthcare practice to improve or prevent care transitions. An overview of the included articles is provided in Table 2. Almost half of the studies (*n* = 12) originated from the USA (40–51), followed by five from China (52–56) and one from India (57), the UK (58), Spain (59), and Australia (60). Seven AI tools were implemented to support the care transition pathway from hospital to home (*n* = 7) (40, 43, 44, 56–58, 60). Six focused on the transition between different levels of care within the same hospital, such as the general care ward, Intensive Care Unit (ICU), and Emergency Department (ED) (41, 45, 50, 51, 55, 59). Three AI tools focused on transitions between the hospital and non-home or outer-hospital settings (46, 53, 54), hence aiming to predict care transitions. Four papers focused on the pathway between a hospital and another hospital (47–49, 52), and one on Emergency Medical Service (EMS) vehicles to ED/hospital (42). A wide range of study designs were present, with the majority having an experimental design used by 40% of all the included articles. Study samples across most articles were discharged patients (*n* = 6) (40, 41, 43, 46, 56, 60), healthcare professionals (*n* = 2) (42, 57), or patients' EHR (*n* = 6) (41, 45, 46, 50, 54, 55). Only one study included family or informal caregivers by integrating their perspectives via experienced feedback to further enhance care continuity (58).

**Table 2:** Characteristics of the 21 included studies.

Author(s), year, country	Study objective	AI^a^ tool for TC^b^/Care transition pathway	Study design and methods	Setting/study population	Study results
Brown et al. 2023, USA (40)	Evaluation of a TC delivery model with AI insights from AI tool Jvion CORE applying social determinants of health data to minimize hospitalization in older adults	Jvion CORE, AI algorithms combined with machine learning > Hospital to Home	Design: retrospective case-control study. Method: using Poisson regression; comparison between TC management enrollers that used AI insights and those without	Adult patients discharged from integrated health system between November 1, 2019—February 31, 2020, and enrolled in a rehospitalization reduction TC management program	Less incidence of 30-day post discharge rehospitalisation Cost-effective Improvement of recovery
Charkoftaki et al., 2023, USA (41)	Using an AI-powered triage platform for future viral outbreaks to detect disease severity and length of hospitalization	AI-powered patient triage system >ED^c^ to ICU^d^	Design: clinical cohort study Method: combination of untargeted metabolomics on plasma data and clinical and comorbidity data to build patients’ triage platform including clinical decision tree, estimation of patient hospitalization length, prediction of disease severity and need of patient transfer	431 participants overall, plasma samples collection from SARS-CoV-2-infected patients during hospitalization (*n* = 111) and from healthy hospital healthcare workers (control group) (*n* = 324)	Higher support in pre-hospital process Earlier prediction of care transitions possible Triaging and classification of patients’ condition more effectively when arriving to ED
Heard et al., 2019, USA (42)	An automatic clinical procedure detection system that uses wearable sensors, video, and machine learning to recognize clinical procedures within emergency services	Automatic clinical detection system >EMS^e^ vehicle to ED/hospital	Design: experimental study Method: Centre for Experiential Learning & Assessment lab at Vanderbilt University served as the data collection environment and contained the clinical procedure equipment	Four participants with varying levels of medical training completed the evaluation; they completed each procedure multiple times within a three-hour timeframe.	Reducing information loss Better information flow for receiving hospitals of the patient's triage level Improvement of patients’ health outcomes Further training needed before realization in real-world
Hewner et al., 2018, USA (43)	Improving post-discharge situation by a nurse care coordinator using innovative technology	Three technology and big data innovations combined in one intervention (care transition alert HEALTHeLINK, ePCAM as clinical decision support, COMPLEXedex as clinical algorithm for big data analysis) >Hospital to Home	Design: quasi-experimental study Method: before and after comparison of the intervention site with resembling primary care practice sites using population-level data	Discharged patients (roster of 6,000 patients) with former chronic diseases	Avoiding readmissions and inpatient and ED visits possible; higher outpatient visits Enhancement of individual patients’ experience Enhancement of work life for healthcare providers Generating revenue due to additional outpatient visits
Jana et al., 2020, India (57)	Using a smartphone-based point-of-care system diagnoses via a machine learning framework	Smartphone-based portable continuous-wave Doppler ultrasound system >Hospital to Home	Design: experimental study/validation study (device development) Method: data acquisition and signal processing	Device examination on 18 volunteers	Improve the outcomes of Point-of-Care testing (resource-limited areas) Baseline for early assessment and detection Faster, cost-effective outcomes
Khanbhai et al., 2022, UK (58)	Providing patient experience feedback via FFT (friends and family test) using natural language processing and machine learning (ML)	NLP (natural language processing) and ML extracting information from FFT >Hospital to Home	Design: retrospective observational study Method: combination of NLP and ML are explored using free text fields identifying favorable services and improvement areas by extracting data from 69,285 FFT reports across four care settings	At a large NHS Hospital in London, which delivers care to around 1 million patients	Variation of patient experience in each service setting Enhancement of patient experience Issues with care transitions identified Shift of resources, previously for manual analysis, now allocated to quality improvement
Long et al., 2017, China (52)	Development of an AI platform for multihospital collaborative use of rare diseases management e.g., Congenital Cataracts (=CC)	AI-platform using deep learning, and involving convolutional neural networks for diagnostics >Hospital to Hospital	Design: multi-hospital clinical trial; website-based study Method: “Finding a needle in a haystack” test, in silico test	The training data set included 410 ocular images of CC of varying severity and 476 images of normal eyes from children, derived from routine examinations	Better interoperability due to cloud-based multihospital AI platform Faster notification to patients and collaborative hospitals
Ortiz-Barrios et al., 2023, Spain (59)	Developing an AI and data-analytics-based framework to predict the likelihood of patients requiring treatments in ICU departments in a pandemic situation	AI and data-analytics-based framework >General ward to ICU	Design: case study Method: an integrated approach for upgrading the intensive bed inventory management	Approach validated in a Spanish hospital	Reduction of waiting time for patients with intervention resulting in a better patient experience Transferring patients to a satellite ICU when internal ICU is overcrowded
Romero-Brufau et al., 2020, USA (44)	Decreasing unplanned hospital readmissions through the use of AI-based clinical decision support	AI-based clinical decision support >Hospital to Home	Design: observational study with a pre-post intervention comparison Method: implementation of an AI tool in one hospital, similar hospitals without AI tool used as a comparison	Assessment of 2,460 hospitalizations	Decreasing readmission rates, especially for high-risk subgroups Minimization of quality gaps when linked with patient-centered intervention
Rubin et al., 2018, USA (45)	Development of a data-driven pediatric early deterioration indicator with purpose of predicting encounters where transfer from general ward to ICU is likely	Automated early deterioration algorithm >General ward to ICU	Design: observational study Method: employing machine learning techniques, and adaptive boosting using electronic health record (EHR) from medical facilities	Use of EHR data collected over 5.5 years from two medical facilities	Improvements in accuracy, sensitivity, and specificity Improvements in the process of ICU transfer, better accuracy Closer monitoring after transfer
Stopa et al., 2019, USA (46)	Prediction of nonroutine discharge after elective spine surgery may improve overall clinical management	ML algorithm predicting risk of non-home discharge for patients undergoing spine surgery >Hospital to Non-home setting (discharge to any setting other than home such as a skilled nursing facility or inpatient rehabilitation).	Design: external validation study Method: Transparent Reporting of a Multivariable Prediction Model for Individual Prognosis or Diagnosis (TRI-POD)	144 patients, who underwent elective inpatient surgery for lumbar disc disorders; Medical records from elective inpatient spine surgery for lumbar disc herniation or degeneration in the Transitional Care Program at Brigham and Women's Hospital (2013–2015)	Via ML algorithm identifying patients at risk of nonroutine discharge Fewer healthcare costs if nonroutine discharge was avoidable
Sun et al., 2021, China (53)	Proposition of a learning framework FedIO bridging Inner- and Outer-hospital information via vertical Federated Learning for perioperative complications prognostic prediction	Learning framework FedIO >Hospital to Outer-Hospital settings (can include home)	Design: experimental study Method: comparison with cloud-based methods	Setting tested inner- and outer-hospital information; comprehensive experiments are derived from real-world datasets	Simultaneous use of inner- and outer-hospital information will increase the accuracy of the predictive model of postoperative complications By predicting complications, faster reactions possible leading to better health outcomes
Xiong et al., 2019, China (54)	Proposing a framework based on deep learning methods for automatic discharge summary generation	Automatic discharge summary generator >Hospital to Outer-Hospital settings (can include home)	Design: experimental study Method: collection of EMRs (Electronic Medical Records) of one-time hospitalization	1,038 patients’ EMRs from a Tier 3A hospital in China	Prediction of discharge diagnosis and medicine care plan possible Effective time-saving for physicians
Xu et al., 2017, China (55)	Addressing the problem of predicting patient flow from longitudinal EHR within Care Units (=CUs) by developing a novel framework for modelling patient transitions among CUs	Predicting the “patient flow” from EHRs via existing ML techniques >ED/ICU to CU	Design: experimental study Method: proposing a mutually-correcting point process interpretation of multinomial logistic regression model to describe the transitions among CUs and the durations in them respectively	Insights in the patient's EHR; real-world data from MIMIC II database; 30,685 patients staying in CUs are selected for training and testing. CUs are categorized into eight departments	Shortening length of stay Reducing preventable readmissions Superior performance in terms of accuracy of predicting CU transition and duration of CU occupancy
Zisis et al., 2020, Australia (60)	Evaluation of the effectiveness of a novel, nurse-led Heart Failure (HF) Disease Management Program (DMP) in selected patients at high risk of short-term hospital readmission, using ultrasound-guided diuretic management and AI to improve HF knowledge in an outpatient setting	Novel DMP using AI to improve knowledge >Hospital to Home	Design: randomized controlled trial Method: prospective multisite randomized controlled trial; comparison with usual care	Allocation of 404 patients hospitalized with acute decompensated HF and ≥33% risk of readmission and/or death after 30 days	AI-based training program providing and enhancing education and patient engagement Through AI integrated avatar-based HF app, a digital coach provides training that prompts appropriate self-care Reducing readmissions
Field et al., 2023, USA (48)	Evaluation of the implementation of an AI-based LVO (large vessel occlusion) detection platform (Viz.ai) on the ischemic stroke workflow, and patient transfer times between outlying (spoke) hospitals and a main regional comprehensive stroke center (hub) for patients undergoing mechanical thrombectomy	Viz.ai which is an AI-powered platform that automatically detects suspected LVOs on CT angiography scans using deep learning algorithms and sends real-time alerts to the stroke team via smartphones >Hospital (spoke) to other Hospital (hub)^f^	Design: retrospective cohort study Method: comparison of pre- and post- implementation of the Viz.ai smartphone application	A total of 262 patients with ischemic stroke and treated with mechanical thrombectomy between Jan 2020—Mar 2022 at a single academic comprehensive stroke center (hub)	Reduction in door-to-puncture time (by 15 min) post-Viz.ai implementation at the comprehensive stroke center (hub) Reduction in transfer time from Viz.ai implementing spoke hospitals (by 37 min) Timely notification of care team and improvement in care coordination and treatment initiation (improvement of the ischemic stroke workflow across the health system/spoke-hub hospital)
Bonner et al., 2024, USA (47)	Evaluation of the implementation of an AI-based image interpretation and communication platform (Viz LVO) across a hospital network on the rate of stroke patient transfers between 5 spoke hospitals and one main/hub comprehensive stroke center and then receiving endovascular thrombectomy and to examine the financial impact of transfer decision-making on hospital and payor costs	Viz LVO (by Viz.ai) an AI-based image interpretation and communication platform, based on an algorithm that is used to identify LVOs on CT angiography and then automates CT angiography image interpretation and enables inter-hospital image sharing and messaging between providers across sites (spoke-hub) > Hospital (spoke) to other Hospital (hub)	Design: retrospective cohort study Method: comparison of two cohorts based on exposure status to AI-system implementation of the Viz LVO across the spoke hospitals (pre-AI cohort and post-AI cohort)	162 pre-AI cohort ischemic stroke patients and 127 post-AI cohort at five spoke community hospitals affiliated with a hub comprehensive stroke center	Enhanced clinical decision-making to assess necessity of patient transfer (increased rate of spoke-to-hub transfer and provision of treatment) Enhanced clinical decision-making to decrease unnecessary transfers to the hub and providing cost savings to the payors (insurer or patient) and higher revenue for the spoke hospitals
Frost et al., 2024, USA (49)	Evaluation of the implementation of an AI-based image interpretation and communication platform (Viz LVO) on the speed for patient transfer, assessment, and delivery of treatment needs	Viz LVO (by Viz.ai) an AI-based image interpretation and communication platform, based on an algorithm that is used to identify LVOs on CT angiography and then automates CT angiography image interpretation and enables inter-hospital image sharing and messaging between providers across sites (spoke-hub) > Hospital (spoke) to other Hospital (hub)	Design: single-hub-and spoke network (8 spokes) retrospective registry analysis Methods: comparison of 6-month periods before and after Viz LVO implementation	132 patients with intracranial LVO (58 pre-Viz LVO and 74 post-Viz LVO implementation) treated with endovascular therapy at a comprehensive stroke network comprising 8 spoke hospitals and 1 hub center	Reduction in time to treatment at the spoke hospitals Significant benefit and reduction in time to treatment for the spoke-to-hub transfers
Hartman et al., 2024 USA (50)	Development of an LLM-generated emergency medicine (EM)-to- inpatient (IP) handoff notes and evaluation of its accuracy and safety compared with physician-written notes	LLM-based tool that automatically generates EM-to-IP handoff notes and clinical summaries >EM to Inpatient ward	Design: retrospective cohort study Methods: comparison of LLM-generated EM handoff notes to physician-written ones	1,600 EM patient records at one medical center	LLM-generated handoff notes exhibited a promising quality comparable to physician-written notes, with no risk to patient safety and overall clinical acceptability
Mayampurath et al., 2025, USA (51)	Evaluation of the implementation of pCART (a machine learning-based pediatric early warning score) and its ability to predict risk of ward to ICU transfer and its association with lower prevalence of critical illness among hospitalized children	pCART (Pediatric Calculated Assessment of Risk and Triage), a machine learning model embedded with the electronic health record system and predicts the risk of patient transfer from ward to ICU within 12 hours > Pediatric hospital ward to ICU	Design: pre- vs. post-implementation retrospective cohort study Methods: comparison of two cohorts pre-implementation/baseline and post implementation for critical event occurrences within 12 h	Pediatric patients below <18 yr. pre-implementation (*n* = 2,763) and post-implementation (*n* = 3,943) admitted to an urban, tertiary-care, academic hospital	Decrease in critical events occurrence within 12 h among patients identified as high risk with Pcart No significant association with overall hospital and ICU length-of-stay, number of ICU transfers, and time to ICU transfer
Chen et al., 2024, China (56)	Development and validation of an interpretable machine learning (ML) model to predict 90-day readmission or mortality in patients with acute heart failure	ML prediction model to predict 90-day readmission or mortality in patients with acute heart failure >Home to Hospital	Design: retrospective cohort study Methods: retrospective analysis of the cohort for 90-day post discharge outcomes using the prediction model	A total of 1,210 patients with acute heart failure discharged from single-center hospital	Effectiveness of the model was demonstrated in prediction of 90-day readmission or mortality rates

A detailed description of the final total 21 studies included in the review by focusing on indicating the AI tool used, relevant care transition pathway, and the reported overall outcomes upon utilising AI for TC.

a AI, artificial intelligence.

b TC, transitional care.

c ED, emergency department.

d ICU, intensive care unit.

e EMS, emergency services.

f Spoke and Hub model refers to a hospital system of care designed to improve access, coordination, and efficiency especially for time-sensitive or specialized treatments like stroke care; Hub hospital refers to a large, specialized medical center (e.g., a comprehensive stroke center) equipped with advanced resources, expert staff, and the capacity to perform complex procedures such as endovascular thrombectomy and it serves as the central referral site in the network; Spoke hospital refers to a smaller or community-based hospital that initially evaluates and stabilizes patients and typically has less specialized care capabilities and relies on timely transfer to a hub for advanced care when needed.

### 3.3 Categorisation of AI usage in transitional care

Most of the AI tools used in TC across the studies could be categorised into at least two categories as per Hayes et al. (26) (see Table 3). Sixteen AI tools were utilised within health information system navigation or interoperability to foster the collaborative work of various technical devices. System navigation guided and supported patients through various care services by providing them with the right information and access points. For example, Jana and colleagues (57) introduced a smartphone-based, portable point-of-care system called continuous-wave Doppler ultrasound. This device could diagnose patients with peripheral arterial diseases using a Machine Learning (ML) framework. Then, through Bluetooth, the smartphone application can instantly receive the blood flow spectrogram through an integrated chip, representing a foundation for early assessment and detection. This allowed the patient to be transferred, if needed, based on the information generated. Another AI tool utilised within system navigation and interoperability combines three technologies into one (43). The first technology, “HEALTHeLINK”, functions as a care transition alert to facilitate the interoperable exchange of medical information between the right healthcare providers. Second, “ePCAM”, serves as a clinical decision support technology that incorporates the patients' social and behavioural determinants of health into the EHR for a better interchange among different healthcare settings and providers. The third technology, “COMPLEXedex”, is a clinical algorithm for big data analysis and is able to classify patients based on their chronic diseases and comorbidities. Hence, this combined AI tool provides a care transition alert to the nurse care coordinator in the event that the discharged patients require post-discharge care management in order to avoid rehospitalizations. Viz.ai was another AI tool that was used and helped in interoperability and system navigation across a hospital network and ultimately enhanced timely care transitions (47–49). This AI-powered platform utilizes deep learning algorithms to automatically detect suspected large vessel occlusions (LVOs) on CT angiography scans, and upon detection, it sends real-time alerts to stroke teams via smartphone, accelerating clinical response and treatment for stroke patients. In addition, it has a specific sub function, known as Viz LVO, which supports inter-hospital communication and image sharing between spoke and hub hospitals, enhancing coordination and decision-making for stroke patients (47, 49). Therefore, by streamlining the identification and transfer process for stroke patients, Viz.ai plays a pivotal role in improving care transitions across hospital systems, particularly in time-sensitive stroke care pathways.

**Table 3:** Categories of AI usage in transitional care .

AI^a^ tool	AI usage in transitional care based on Hayes et al. (26)			
	Discharge/follow-up	Triaging/predicting models of care	Interoperability/system navigation	Language translation
AI algorithm called Jvion Core (40)	X		X	X
AI-powered patient triage system (41)		X	X	X
Automatic clinical detection system (42)		X	X	X
Three innovative technologies and Big Data (43)	X	X	X	
Smartphone-based continuous-wave Doppler ultrasound (57)			X	
NLP^b^ & ML^c^ extracting information from FFT^d^ (58)	X			X
AI agent with cloud-based platform (52)		X	X	X
AI and data-analytics-based framework (59)		X		X
AI-based clinical decision support (44)	X		X	
Automated early deterioration algorithm (45)			X	X
ML algorithm predicting risk of nonhome discharge (46)	X			X
Learning framework FedIO (53)	X		X	
Automatic discharge summary generator (54)	X		X	
Modeling patient transitions among CUs^e^ (55)	X		X	X
Novel DMP^f^ using AI improving knowledge (60)	X			X
Viz.ai an AI-powered detection platform (48)	X	X	X	
Viz LVO^g^ (47)	X	X	X	
Viz LVO (49)	X	X	X	
LLM-generated EM-to-IP handoff notes^h^ (50)	X		X	X
pCART^i^ (51)	X	X	X	
ML prediction model (56)	X	X		
*Total number of studies/AI tools*	*15*	*10*	*16*	*11*

Indicating the type and form of usage of each of the 21 AI tools for transitional care. Values in last row in italic present the total number or frequency of studies and AI tools under each category/column.

a AI, artificial intelligence.

b NLP, natural language processing.

c ML, machine learning.

d FFT, friends and family test.

e CUs, care units.

f DMP, disease management program.

g LVO, large vessel occlusions.

h LLM-generated EM-to-IP handoff notes, large-language models generated to emergency medicine to inpatient handoff notes.

i pCART, pediatric calculated assessment of risk and triage.

Eleven AI tools were used in TC for language translation, whereby it appears as Natural Language Processing (NLP) by translating, for instance, the information from EHRs into an automatic text generation (40) or by creating an AI agent with a cloud-based platform for medical data exchange for multi-hospital collaborative usage (52).

Another way of using AI in TC is for discharge and follow-up solutions which was seen for 15 AI tools. Romero-Brufau et al. (44) introduced an AI-based clinical decision support to decrease unplanned hospital readmissions and help discharged patients remain in their assigned healthcare setting. In another study, an AI tool characterised by an ML algorithm was developed to predict patients at risk of nonroutine discharge after elective spine surgery (46). Here, this tool could predict the probability of postoperative discharge to any setting other than home by analysing the multiple patient medical variables and aiming to improve the overall clinical management for these patients. Similarly, Viz.ai was an AI tool that facilitated timely discharge decisions by identifying patients in need of advanced stroke care, enabling their quick transfer from a spoke hospital to a comprehensive stroke center (hub) for appropriate treatment (47–49).

Ten AI tools were used for triaging and predicting models of care. In Charkoftaki et al. (41), an AI-powered patient triage platform consisted of three parts: clinical decision tree, calculation of patient hospitalisation length, and the outlook of the disease's severity with the need for patient transfer to the ICU. Also, Ortiz-Barrios and colleagues (59) developed an AI and data-analytics-based framework to predict the probability of patients requiring treatment within the ICU department during the COVID-19 pandemic, whereby patients were first triaged regarding their urgency and necessity and second models of care could be estimated most fitting for the individual patient, in this case, transferring to ICU to get specific treatment or remaining on the regular ward. Similarly, pediatric calculated assessment of risk and triage (pCART) was a machine learning model AI tool integrated into the electronic health record system, and helped to do early triage and predict the likelihood of a pediatric patient requiring transfer from the hospital ward to the ICU within 12 h (51).

### 3.4 AI tools used for a comprehensive and effective transitional care

Table 4 offers a comprehensive overview indicating which components of TC were addressed by the identified AI tools. Care continuity and complexity management were the two components of comprehensive TC that were promoted most by using AI tools, followed by patients’ and caregivers' well-being and then caregiver engagement. As for the components of accountability and caregiver education, they were promoted by eight and seven AI tools respectively. However, patient engagement and education were the components that appeared to be the least promoted using AI tools. For instance, care continuity for patients as they transition across multiple healthcare settings and care teams was provided via medical information sharing using an AI agent with a cloud-based platform between multiple hospitals, as described in the study conducted by Long et al. (52). This was to ensure the best possible treatment for patients with rare diseases such as congenital cataracts, for which some hospitals might be better equipped. By using information and knowledge sharing through the AI agent integrated into the cloud-based platform, the advice of a transfer to a collaborative, advanced hospital regarding this uncommon disease can occur, facilitating care continuity. Similarly, Xiong et al. (54) generated an automated discharge summary for the outer hospital setting whereby the following responsible care team receives the necessary information on the patient's admission, lab tests, examinations, diagnostic findings, treatments, and medication care plan. Hence, with this automated process of transferring this valuable information, the continuity of patient care can be enhanced without wasting financial and temporal resources on double examinations.

**Table 4:** AI tools mapped to the components of comprehensive transitional care.

AI^a^ tool	Components of comprehensive transitional care							
	Patient engagement	Caregiver engagement	Complexity management	Patient education	Caregiver education	Patient and caregiver well-being	Care continuity	Accountab-ility
AI algorithm called Jvion Core (40)							X	X
AI-powered patient triage system (41)			X		X	X		
Automatic clinical detection system (42)			X		X	X	X	X
Three innovative technologies and Big Data (43)		X			X	X	X	X
Smartphone-based continuous-wave Doppler ultrasound (57)	X	X	X	X				
NLP^b^ & ML^c^ extracting information from FFT^d^ (58)					X	X	X	
AI agent with cloud-based platform (52)		X	X			X	X	
AI and data-analytics-based framework (59)		X					X	
AI-based clinical decision support (44)		X			X	X	X	
Automated early deterioration algorithm (45)						X	X	X
ML algorithm predicting risk of nonhome discharge (46)				X	X		X	X
Learning framework FedIO (53)	X	X	X				X	
Automatic discharge summary generator (54)		X	X			X	X	X
Modelling patient transitions among CUs^e^ (55)		X				X	X	X
Novel DMP^f^ using AI improving knowledge (60)	X	X	X	X	X	X	X	
Viz.ai an AI-powered detection platform (48)			X				X	
Viz LVO^g^ (47)			X				X	
Viz LVO (49)			X				X	
LLM-generated EM-to-IP handoff notes^h^ (50)							X	X
pCART^i^ (51)			X				X	
ML prediction model (56)			X				X	
*Total number of studies*/*AI tools*	*3*	*9*	*12*	*3*	*7*	*10*	*19*	*8*

Indicating for each of the 21 AI tools, which components of transitional care it addressed. Values in last row in italic present the total number or frequency of studies and AI tools under each category/column.

a AI, artificial intelligence.

b NLP, natural language processing.

c ML, machine learning.

d FFT, friends and family test.

e CUs, care units.

f DMP, disease management program.

g LVO, large vessel occlusions.

h LLM-generated EM-to-IP handoff notes, large-language models generated to emergency medicine to inpatient handoff notes.

i pCART, pediatric calculated assessment of risk and triage.

Additionally, Heard et al. (42) outlined in their research the enhancement of current EMS communication flow with the receiving hospital by integrating an automated non-invasive system that detects clinical procedures and communicates the patient's triage level. Here, machine learning is employed to identify necessary clinical procedures within emergency services. This approach ensures improved information flow regarding the patient's health status for receiving hospitals, facilitating continuity of care. Also, a novel nurse-led Disease Management Program (DMP) using AI to improve knowledge in an outpatient setting helps ensure a smooth continuation of improving patients' care and overall health (60).

As for enhancing the well-being of patients and caregivers, implementing a nurse care coordinator function utilising three combined AI innovative technologies and big data improved the post-discharge experience within the home environment (43). This approach enhanced patient satisfaction and caregivers' well-being by alleviating the burden of sole responsibility for the patient's health, as well as allowing for a more effective use of healthcare resources.

Furthermore, by developing an AI-based framework to predict the possibility of patients needing ICU transition, patients' and caregivers' well-being improved due to the better-fitting circumstances provided after being transferred to the ICU (59). Khanbhai et al. (58) developed the possibility to provide feedback regarding patient experience using NLP and ML, which helped patients and their informal caregivers better express their needs and know better where improvement is needed, leading to higher well-being on both sides.

Enhancing caregiver engagement as a component of comprehensive TC was possible during the collaborative sharing of accurate diagnoses and treatment decisions developed by an AI agent for patients living with rare diseases from hospital to hospital (52). Similarly, by bridging inner and outer hospital knowledge through the learning framework FedIO, patient information such as complications or prognostic predictions can be made available for both settings by integrating each care team into the treatment procedure (53). Also, a novel framework modelling patient transitions between ED or ICU to a normal Care Unit (CU) helped to engage all caregivers from different wards in the care process within one facility (55).

Twelve studies also identified that AI can be used in TC to manage care complexity better (41, 42, 47–49, 51–54, 56, 57, 60). The AI tool proposed by Sun et al. (53), Xiong et al. (54), and Zisis et al. (60) incorporates the intricacies of medication management into its system. Doing so effectively prevents the transmission of misinformation regarding the regulated use of medications through the automated discharge summary generator, as highlighted by Xiong et al. (54). Also, the medication information is integrated into bridging inner and outer hospital information, decreasing the chance of incorrect medication administration (53). Likewise, Zisis and colleagues (60) AI-enabled DMP utilises a record of all medications currently in use or used in the past to counteract the complexity of medication management. Similarly, Viz.ai and pCART were two AI tools that supported complexity management in patients undergoing critical care transitions. pCART, a machine learning model embedded in the electronic health record, predicted the risk of pediatric patients requiring ICU transfer within 12 h, enabling proactive triage and escalation of care from the normal hospital ward (51). Also, Viz.ai leverages deep learning to detect large vessel occlusions on CT angiography and facilitates real-time communication between hospitals. By enabling timely discharge and transfer from spoke hospitals to comprehensive stroke centers (hubs), Viz.ai streamlined decision-making for stroke patients (47–49). Therefore, both AI tools played pivotal role in complexity management during care transitions by enhancing early clinical awareness and then timely coordination and intervention for critical transitions of care.

The component of caregiver education was found in seven studies (41–44, 46, 58, 60), and was for example characterized by the extra training needed to correctly use an automated detection system (42). Likewise, before educating patients and sharing information about heart failure, caregivers needed to first develop specific knowledge on the AI-enabled nurse-led heart failure disease management program, as demonstrated in the study by Zisis et al. (60).

Accountability was also identified as a component of TC in eight studies (40, 42, 43, 45, 46, 50, 54, 55). As introduced in the study of Hewner et al. (43), a nurse care coordinator was fully integrated into all the care processes an individual patient may pass through. This role was activated by the AI-generated care transition alerts, allowing for timely care management and coordination post-discharge. Thus, instead of the previous and current healthcare setting being responsible for the patient, the nurse care coordinator and the other facilities now share accountabilities regarding the patient's health. Similarly, in the study by Xu et al. (55), the developed framework for modelling patient transitions among different care units such as ED, ICU, or a regular ward, helped determine who is in charge and which care team will be responsible for a specific patient.

On the other hand, patient engagement and patient education were shown to be the least promoted by AI tools for TC. One example was the use of an avatar-integrated app, whereby patients were self-educated on heart failure (60).

Six studies (43, 44, 52, 54, 55, 60) included all three common components namely care continuity, patients' and caregivers' well-being, and caregiver engagement. Notably, the AI tool introduced by Zisis and colleagues (60) in an Australian healthcare setting has seven out of the eight components for promoting a comprehensive and effective TC. This novel nurse-led DMP makes use of various interventions, among others integrating a digital coach into an avatar-based app, whereby the coach provides interactive tasks and AI-guided education can be examined based on the patient's answers. According to Naylor et al. (14), healthcare systems must aim to encompass all eight components to guarantee an optimal TC experience for patients, whereby the degree of attention between components can vary depending on the unique requirements both patients and caregivers might have.

### 3.5 Outcomes of AI tools used for transitional care

The reported outcomes and impact of AI tools in TC, retrieved from the 21 studies, were diverse, heterogeneous, often overlapping, and in some cases represented estimations or predictions rather than direct measurements. Thus, due to this variability across the studies, meaningful comparisons or synthesis of a combined effect was not feasible. Consequently, a comprehensive mapping of the reported outcomes was conducted, through which seven outcomes were identified and described as being associated with enhancing transitional care delivery. These included: rehospitalization rates, earlier prediction and diagnosis, patients' health outcomes, information exchange, patient experience, cost-effectiveness, and care transition performance (see Table 5).

**Table 5:** Reported outcomes of AI in transitional care.

AI^a^ tool	Reported Outcomes per each AI tool						
	Rehospitalization rates	Earlier prediction/earlier diagnosis^j^	Patients’ health outcomes	Information exchange	Patient experience	Cost-effectiveness	Care transition performance
AI algorithm called Jvion Core (40)	X		X			X	
AI-powered patient triage system (41)		X					
Automatic clinical detection system (42)		X	X	X			
Three innovative technologies and Big Data (43)	X					X	
Smartphone-based continuous-wave Doppler ultrasound (57)		X				X	
NLP^b^ & ML^c^ extracting information from FFT^d^ (58)			X		X	X	
AI agent with cloud-based platform (52)	X		X	X			
AI and data-analytics-based framework (59)	X				X		X
AI-based clinical decision support (44)	X		X	X			
Automated early deterioration algorithm (45)			X				X
ML algorithm predicting risk of nonhome discharge (46)	X			X		X	
Learning framework FedIO (53)		X	X	X			
Automatic discharge summary generator (54)		X		X			
Modeling patient transitions among CUs^e^ (55)	X	X		X			
Novel DMP^f^ using AI improving knowledge (60)	X		X	X	X		
Viz.ai an AI-powered detection platform (48)		X		X			X
Viz LVO^g^ (47)		X		X		X	X
Viz LVO (49)		X		X			X
LLM-generated EM-to-IP handoff notes^h^ (50)				X			
pCART^i^ (51)		X	X				X
ML prediction model (56)	X	X					
*Total number of studies*/*AI tools*	*9*	*11*	*9*	*12*	*3*	*6*	*6*

Indicating for each of the 21 AI tools, which outcomes were reported upon its usage for transitional care. Values in last row in italic present the total number or frequency of studies and AI tools under each category/column.

a AI, artificial intelligence.

b NLP, natural language processing.

c ML, machine learning.

d FFT, friends and family test.

e CUs, care units.

f DMP, disease management program.

g LVO, large vessel occlusions.

h LLM-generated EM-to-IP handoff notes, large-language models generated to emergency medicine to inpatient handoff notes.

i pCART, pediatric calculated assessment of risk and triage.

j Earlier prediction equals earlier prediction of pathways, whereas earlier diagnosis equals earlier diagnosis for a disease.

#### 3.5.1 Rehospitalization rates

Nine studies reported on rates of rehospitalization following patient discharge upon the application of AI tools for transitional care (40, 43, 44, 46, 52, 55, 56, 59, 60). For example, less incidence of 30-day post-discharge rehospitalization (21.0% lower adjusted incidence of 30-day rehospitalization rate) after once released was identified as one of the main outcomes of using the AI tool Jvion CORE, besides improvement of recovery and increased cost-effectiveness due to saving the average cost of any additional rehospitalization (40). Hewner et al. (43) found that implementing a care transition alert combined with clinical decision support and a clinical algorithm for big data analysis coordinated by a nurse functioning as a care coordinator will avoid readmissions and emergency department visits. This was demonstrated by a 25% reduction in inpatient visits and a 35% decrease in emergency department visits compared to baseline or usual rates. Similarly, in the implementation of an ML prediction model to predict 90-day readmission rate in a cohort of 1210 patients, 28.4% experienced readmission or mortality within 90 days post-discharge (56).

#### 3.5.2 Earlier prediction and diagnosis

In 11 studies, earlier predictions or diagnoses were identified as potential effects AI can have when integrated into TC (41, 42, 47–49, 51, 53–57). By utilising an AI-powered triage platform introduced by Charkoftaki and colleagues (41), earlier predictions of care transitions were possible by detecting the disease severity or the length of hospitalisation, while the automated clinical detection system from Heard et al. (42) holds the possibility of an earlier diagnosis of patients in the EMS vehicle and transferring the gained knowledge to the receiving hospital before the patient arrives. Similarly, both AI tools Viz.ai and pCART could help in the early detection of critical patient conditions and provide early awareness and timely alerts to the care team about anticipated care needs (47–49, 51). Thus, this allowed for a timely notification of care team, and improved care coordination (47–49), as well as a decrease in critical events occurrence within 12 h among patients (51).

#### 3.5.3 Patients' health outcomes

Integrating AI tools into routine TC showed a significant increase in the patients' health outcomes in nine studies (40, 42, 44, 45, 51–53, 58, 60). For instance, Sun et al. (53) introduced a learning framework named “FedIO”, designed to predict complications and enable faster response times, potentially leading to improved patients' health outcomes. Additionally, integrating an AI-based clinical decision support systems has led to a notable reduction in readmission rates, particularly among high-risk subgroups (44). This decline in quality gaps ultimately contributes to patients' overall well-being.

#### 3.5.4 Information exchange

Ensuring a continuous and timely flow of patient medical information between providers and settings was the most common reported outcome of AI tools when applied for TC as described in 12 studies (42, 44, 46–50, 52–55, 60). This was particularly evident when medical data was transferred between hospitals, allowing for the collaborative and quick development of the optimal treatment solution and sharing knowledge throughout the transition process (47–50, 52). For example, when patients were discharged from a hospital to an outer-hospital setting, such as home, a rehabilitation center, or a nursing home; an automated discharge summary could save time for the physician in charge of the ongoing care process. This was achieved through the direct transfer of the patient's medical information and follow-up details on prior treatments (54).

#### 3.5.5 Patient experience

A better patient experience was reported as an outcome in three studies (58–60). This was demonstrated when issues within care transitions could be identified via the provision of a patient experience feedback tool using NLP and ML to extract information (58), when patient waiting times were reduced to enhance the patient flow based on an AI and a data-analytical framework (59), or when an AI-based training program was provided; which enhanced patient education and the overall experience (60).

#### 3.5.6 Cost-effectiveness

Six studies highlighted the potential cost-effectiveness of applying AI tools in transitional care settings by reducing avoidable healthcare expenditures (40, 43, 46, 47, 57, 58). In the study by Brown et al. (40), the use of the Jvion CORE AI tool was associated with reduced rehospitalizations and unplanned acute care utilization, leading to projected savings by avoiding the average costs associated with additional hospital stays. Similarly, Hewner et al. (43) reported a reduction in per capita costs among patients with chronic diseases. The AI tool used here was estimated to save a $664 per patient through avoided healthcare encounters and reduce inpatient and emergency department utilization by $1,669 per patient in comparison to a previous year's baseline rates. This was also suggested in the study by Stopa et al. (46), whereby machine learning algorithms could help avoid nonroutine discharges, potentially reducing overall healthcare costs. On the other hand, Jana et al. (57) emphasized the cost-effectiveness of AI-enabled tools when applied in resource-limited settings, where machine learning and Bluetooth-enabled data transmission facilitated timely patient triage and transfer decisions. Likewise, Viz LVO could enhance the clinical decision-making process of the care team and help to decrease unnecessary transfers to the hub hospitals, which was potentially estimated to provide cost savings to the payors (insurer or patient) and higher revenue for the spoke hospitals if they provided the treatment instead of the hub (47).

#### 3.5.7 Care transition performance

Care transition performance emerged as a multi-dimensional outcome across six studies, evaluated through transfer rates between settings, time to treatment, and time to transfer (45, 47–49, 51, 59). While one study found no significant change in the number or timing of ICU transfers from hospital wards upon applying the pCART risk and triage tool (51), two other studies demonstrated measurable improvements following Viz.ai implementation. One reported a reduction in time to treatment at spoke hospitals and during transfers to hub hospitals (49), while another showed enhanced decision-making that led to increased rate of spoke to hub necessary transfers and treatment provision (47). Also, one study revealed a 37 min reduction in transfer time from a Viz.ai implementing spoke hospital to the hub; as well as a 15-minute decrease in time to treatment initiation when the patient arrives at the hub (48). In another two studies, the AI tools used supported the decision-making and accuracy to improve the transfer process to ICU settings (45, 59). Collectively, these findings underscore the potential of AI tools to optimize the timing and appropriateness of care transitions between settings.

#### 3.5.8 Adverse outcomes

 On the other hand, adverse outcomes were only identified in two studies such as the extra training required to understand and be able to use and interpret an automated detection algorithm efficiently (45). Likewise, the need to prove cost-effectiveness by using earned revenue on expenditures like the AI tool itself or to support the broadened role of the nurse care coordinator financially (43). Although these unfavourable effects exist, they were not as distinctive as the more promising ones.

## 4 Discussion

This scoping review addresses key knowledge gaps regarding the application of AI in transitional care based on findings from 21 studies. It identifies a variety of AI tools designed to support, improve, or prevent transitions of patients across different care settings.

Most tools focused on transitions from hospital to home, followed by hospital to other care facilities, intra-hospital transitions, hospital-to-hospital, and emergency unit to hospital admissions. The AI tools were primarily used for discharge and follow-up, language translation, and system level interoperability and system navigation. These included technologies such as algorithms incorporating machine learning into AI platforms using deep learning or automated discharge generators. Among the components of comprehensive and effective TC that could be most supported by AI tools were care continuity, complexity management, patient and caregiver well-being, and caregiver engagement. Notably, care continuity was the most frequently targeted component. Conversely, patient engagement and education appeared to be less promoted by AI tools. Earlier prediction and diagnosis and information exchange were prominent outcomes reported when applying AI for TC. Moreover, the use of AI tools to support care continuity has shown promising outcomes, including reduced rehospitalization rates, continuous information flow, and overall improvement in patients' health. These outcomes highlight the promising potential of AI for enhancing TC.

The current review demonstrated that AI has the potential to play a significant role in further improving TC, especially by supporting those responsible for organizing TC. This resonates with the recent literature on the frequent application of AI in healthcare including applications for patient engagement, administrative efficiency, diagnosis and treatment selection (61). Specifically, AI's function to support TC services can be designated as redistributing tasks among healthcare professionals and streamlining their workloads (62). This was reported in the literature as instrumental to circumvent unnecessary hospital admissions through early diagnosis and screening, providing tailored medical advice for chronic disease management, and diverting patients to the appropriate healthcare pathway (62). Therefore, literature highlights the need for AI-enabled applications to be embedded more deeply into the daily practice of those responsible for delivering TC (25). AI tools are seen as promising when applied to services affecting patients directly, whereby ML algorithms and deep learning features prove to be more useful in enhancing the accuracy of diagnostics made by physicians and predicting the patient's prognosis (63). Consistent with this, our review found AI useful in predicting diagnosis as well as the risk of rehospitalizations for patients. In both cases, this helps save time by establishing this information early on and improving care transitions. Similar applications were also reported in the literature, whereby AI tools were used for the diagnosis, outcome-prediction, and management of heart failure (64), and the early identification and prediction of patients at risk of sepsis (65). On the other hand, research by Kuwaiti et al. (66) indicated that AI tools with deep learning algorithms can sometimes be less capable of providing substantial comprehensive justifications for forecasts.

While many AI tools may not be explicitly designed for transitional care, several applications identified in the broader healthcare literature may still indirectly enhance TC pathways. Diagnostic support systems, remote self-management technologies, early warning scores, and chronic disease management algorithms can improve the timing of transitions by enabling earlier recognition of deterioration or recovery (67, 68). Likewise, predictive models for chronic conditions such as heart failure may strengthen coordination and continuity by informing more proactive handovers, preventing unplanned transitions, or ensuring that patients are routed to the appropriate level of care (68). These indirect effects suggest that future evaluations of AI in TC should adopt a wider systems perspective, acknowledging that upstream diagnostic or monitoring tools can meaningfully and ultimately shape transitional care processes.

Importantly, our findings highlight that most of the current application of AI in TC is oriented toward supporting healthcare professionals through administrative and operational efficiencies, rather than replacing humans with AI. Across the studies reviewed, AI tools were predominantly used for tasks such as system navigation, information exchange, managing follow up processes, and enhancing communication, functions that reduce administrative burden. Only a minority of AI applications address complex cognitive tasks, such as predictive modelling for patient trajectories. These findings show that AI is not yet a substitute for human intelligence, but rather a tool to enhance safety, coordination and efficiency within TC. While AI tools have shown promise in improving outcomes, it is often difficult to disentangle their effect of the technology from the expertise of the clinicians who use them. For example, in systems where nurse managers use AI to support discharge planning or care coordination, improvements may stem as much from the enhanced decision-making and workflow of the clinician as from the AI itself. This highlights the importance of viewing AI as a tool that augments, rather than replaces, human judgment; particularly in complex, context-sensitive areas like transitional care. Nevertheless, the performance prospects of AI tools in TC in terms of cost-effectiveness remain uncertain due to the initial high acquisition costs (69). However, this review found that while AI tools used for TC appear to be only partially cost-effective, using the revenue gained after being amortised can still be cost-effective. A study by Areia et al. (69) demonstrated similar findings, whereby AI-enabled screening tools can be a cost-saving strategy compared to the traditional methods and can help to further prevent the future incidence and mortality of cancer diseases. Likewise, another study showed that AI-assisted follow-up of patients after surgery can save human resources costs (70). A challenge can arise when the financial benefits of deploying AI are unevenly distributed or accrue to parties other than those making the investment. This can hinder the adoption of AI.

While the potential benefits of AI in enhancing healthcare services, including transitional care, are promising, there are notable drawbacks and challenges associated with its implementation (71). First, the empirical evidence regarding AI's positive impact, particularly on patient outcomes, remains limited, which may impede its adoption by healthcare organisations. Second, the allocation of resources such as time, costs, and training required for AI implementation poses significant considerations (62). Third, there are rising concerns regarding user acceptance and the potential risk of overreliance on AI tools in decision-making processes (72). Moreover, the impact of integrating AI into healthcare professionals' workflows could lead to resistance. It was reported that some may experience AI as threatening, obstructive, impeding, or biased in making clinical decisions (62, 72). Fourth, ethical issues including privacy, accountability, and regulation for using AI tools in healthcare are commonly reported implementation challenges (73). In a recent study about the patient's perspectives on the use of AI, concerns related to the safety of AI and threats to the patient's choice and autonomy were reported (74). This aligns closely with care transitions, emphasizing that patient preferences, such as their choice to remain at home rather than move to a nursing home, should be taken into account irrespective of the AI tools utilized for care transition decisions.

Additionally, there is a growing concern that using AI can lead to deskilling or even the replacement of healthcare professionals (75). Whether this could be an intended or unintended consequence of AI, it requires attention and further guidance to ensure that the knowledge, skills, and clinical experience of healthcare professionals are retained while leveraging AI. Therefore, the focus should not be on whether AI can be used in healthcare or not but rather on how. Hence, as recommended by Aquino et al. (76), non-care tasks can be more suitable for automation by AI, while those that require human touch and can generate better outcomes upon the direct engagement of patients with healthcare providers should be kept for humans. In turn, this allows for preservation of both the relational and care aspects of healthcare services. Furthermore, to mitigate deskilling of healthcare professionals, it is imperative to develop AI training systems as well as keep certain tasks, although mundane, to be performed by professionals in order to retain their skills (76). This also resonates with the work by Raisch et al. (77), indicating the need to balance AI-driven automation (replacing human tasks) and augmentation (enhancing human work), arguing that both are interdependent rather than mutually exclusive. Thus, it highlights the importance of organizations maintaining a balance between the two functions to fully harness AI's potential while retaining human value and capacity in decision-making and creativity. Therefore, despite opportunities for using AI in transitional care, several important gaps remain, particularly when viewed through the lens of the automation-augmentation paradox (77). Current AI applications tend to address isolated components of TC, such as discharge planning or readmission prediction, without supporting the end-to-end coordination that continuity of care requires. This fragmented integration limits their contribution to patient outcomes. Moreover, many tools privilege automation over augmentation, streamlining administrative workflows but offering limited support for the clinical reasoning and contextual judgment that clinicians bring to transitional decisions. As a result, AI risks displacing rather than strengthening human expertise. Equity concerns further exacerbate these shortcomings whereby models trained on narrow datasets, or tools that inadequately support cultural and linguistic diversity, may amplify inequalities in access and outcomes (30). Advancing the field, therefore, requires a shift toward human-centered and equity-driven support. This includes developing explainable and adaptive AI that learns from clinician feedback and designing interoperable systems that connect hospital, primary, and community care. Such innovations would move AI in TC beyond task automation toward really supporting clinicians and patients in navigating complex transitional pathways.

On the other hand, this review identified several outcomes that can be associated with the use of AI in transitional care, highlighting the potential impact on patient experiences and care processes. However, it is important to recognize that there are currently no standardized approaches to assessing the role of AI tools in transitional care. This creates challenges in evaluating their effectiveness and comparing outcomes across studies. Hence, this study represents a new step towards developing an evaluation framework that can be built upon as AI tools continue to proliferate across the healthcare sector. By systematically identifying and analysing the outcomes of AI implementations in transitional care, we aim to contribute to the knowledge base needed to develop a standardized evaluation framework. Such a framework could guide future research, inform best practices, and facilitate the integration of AI tools into transitional care settings, ultimately improving patient outcomes and quality of care. In line with a published model for evaluation of AI in healthcare (78), a framework could include key elements such as clinical relevance and fit for transitional care (e.g, readmission rates, post-discharge mortality, number and frequency of care transitions), patient usability (e.g., accessibility, patient adherence rates), cost-effectiveness, equity (e.g., AI application for TC across diverse populations), early diagnosis/prediction accuracy and reliability for care transitions, and compatibility of AI application for TC with the existing healthcare system.

In the future, it will be essential to encourage collaboration between researchers, practitioners, and policymakers to establish standardized criteria for evaluating AI tools in transitional care. This collaborative effort will not only promote consistency in assessments, but also help identify best practices and areas for improvement as AI technologies evolve.

## 5 Limitations

This review has some limitations. First, the aim was to review the scientific literature, and hence opted to exclude grey literature. However, it is worth noting that grey literature could potentially offer valuable insights into AI advancements within transitional care. Additionally, while our search strategy included three major databases, we may have excluded relevant studies from other sources. Second, even though an extensive search strategy was used to identify relevant studies for AI tools used in TC, some other relevant research could have been missed, as sometimes the study aim of AI tools in TC is not always clearly described. Third, the inclusion and exclusion criteria may have introduced selection bias, potentially excluding relevant studies that did not meet the predefined search terms or database parameters. This could result in an incomplete representation of the field. Fourth, the review may be subject to publication bias, as studies reporting positive outcomes or successful AI implementations are more likely to be published, while negative or inconclusive findings may remain underreported. Lastly, it is important to note that AI is a rapidly evolving field, which is why this scoping review has identified only 21 studies. Therefore, this is an overview of AI tools in TC identified between years 2013 and 2025, while we are in the early stages of using AI in transitional care. This reflects the early stage of AI adoption in TC, and our findings should be interpreted as an initial overview rather than a definitive assessment. These limitations may influence the overall interpretation of AI's applicability in transitional care, and thus future research can consider broader search strategies and grey literature to mitigate these limitations.

## 6 Conclusions

In the 21 studies examined in this review, the integration of AI in transitional care showed the promise of enhancing care continuity between different settings through organizing the discharge/follow-up processes as well as reinforcing system navigation and interoperability, leading to reduced rehospitalization and better patient information exchange. This review contributes significantly to the imperative need to continually enhance transitional care, which is a critical healthcare service issue with a far-reaching societal impact affecting not only patients but also healthcare providers, organisations, and healthcare systems globally. Moving forward, future research should further explore the intersection of AI and TC, since the implications of identical AI tools implementations can vary across different contexts. Therefore, we advocate for thorough investigations into effective implementation strategies of AI in TC, taking into account situational factors and embedding practices within local settings. Additionally, further empirical research on the performance effects of AI in TC is needed to measure outcomes in practice, ideally by utilizing an established, valid, and consistent evaluation framework. Also, it is essential to address potential challenges associated with AI use in TC by identifying situations where AI may raise privacy concerns and contribute to the dehumanisation of healthcare service delivery (79). As AI and transitional care continue to gain increased attention for their potential synergies in practical settings, research is warranted to explore AI applications across all facets of transitional care.

## Data Availability

All information and data supporting the findings of this study are derived from publicly available literature, which has been appropriately cited within the article. However, data analysis codes and mapping performed during this study are available from the corresponding author upon request.
